# Extracts from Wheat, Maize, and Sunflower Waste as Natural Raw Materials for Cosmetics: Value-Added Products Reaching Sustainability Goals

**DOI:** 10.3390/pharmaceutics16091182

**Published:** 2024-09-07

**Authors:** Milica Lukić, Ana Ćirić, Dragana D. Božić, Jelena Antić Stanković, Đorđe Medarević, Zoran Maksimović

**Affiliations:** 1 Department of Pharmaceutical Technology and Cosmetology, Faculty of Pharmacy, University of Belgrade, Vojvode Stepe 450, 11221 Belgrade, Serbia; ana.ciric@pharmacy.bg.ac.rs (A.Ć.); djordje.medarevic@pharmacy.bg.ac.rs (Đ.M.); 2 Department of Immunology and Microbiology, Faculty of Pharmacy, University of Belgrade, Vojvode Stepe 450, 11221 Belgrade, Serbia; dragana.bozic@pharmacy.bg.ac.rs (D.D.B.); jelena.stankovic@pharmacy.bg.ac.rs (J.A.S.); 3 Department of Pharmacognosy, Faculty of Pharmacy, University of Belgrade, Vojvode Stepe 450, 11221 Belgrade, Serbia; zoran.maksimovic@pharmacy.bg.ac.rs

**Keywords:** harvest residues, waste valorization, maize, wheat and sunflower extract, skincare

## Abstract

Agricultural waste is underutilized, and sometimes burning them has a negative impact on the environment and human health. This research investigates the untapped potential of extracts from maize, wheat and sunflower waste as natural materials for cutaneous, specifically, cosmetic application. The possibility of incorporating lipid and ethanol extracts from wheat, maize, and sunflower into creams was investigated together with their potential contribution to the structural and functional properties of the topical formulations. Results of the physicochemical characterization show that investigated extracts can be successfully incorporated into creams with satisfactory stability. All extracts showed a desirable safety profile and good antimicrobial activity against various microorganisms. Lipid extracts have proven to be promising structural ingredients of the oil phase, contributing to the spreadability, occlusivity, and emollient effect. Ethanol extracts influenced washability and stickiness of the formulation and could be considered as prospective ingredients in self-preserving formulations. The extracts affected the sensory properties of the creams, mainly the smell and color. These results suggest that the extracts from wheat, maize, and sunflower waste could be used as multifunctional natural ingredients for cosmetic formulations which can replace less sustainable raw materials. This also represents a valorization of waste and is in line with broader sustainability goals.

## 1. Introduction

In order to improve quality of life and thus general well-being, people are increasingly turning to holistic approaches that go beyond traditional healthcare. Cosmetic products play an important role in this context. The use of skincare and cleansing products contributes to the health of the skin and the whole body, while daily skincare routines and decorative cosmetics improve body image, self-esteem, and quality of life [1,2]. Interestingly, the aspect of product choice based on personal preference contributes significantly to the quality of life, which refers in particular to the possibility of purchasing products that comply with the principles of sustainability [3,4].

Cosmetics are topical products, i.e., substances or mixtures intended to be placed in contact with the external parts of the human body (epidermis, hair, nails, lips, and external genital organs) or with the teeth and mucous membranes of the oral cavity, mainly to cleanse them, perfume them, change their appearance, protect them, keep them in good condition or correct body odor [5]. These products are used daily in large quantities and most of them are applied directly to the skin, making them one of the most important cutaneous or topical products among general products [6]. At the same time, these products are a source of exposure to a large number of chemicals, which are important for human safety as well as environmental safety [7,8]. Despite the existing regulations for cosmetic products that aim to ensure their safety for humans and the environment, there is a growing demand for safer and sustainable cosmetic products and raw materials [9]. In recent years, not only the cosmetic but also the pharmaceutical industry has undergone a swift transformation, striving to develop natural, organic, and environmentally friendly formulations, with an increasing focus on responsible and ethical sourcing of raw materials, promoting fair trade practices, reducing emissions and waste, using recycled and recyclable packaging, and increasing the proportion of biodegradable ingredients in formulations [10,11]. Not surprisingly, plants are the main source of new raw materials for these formulations [12,13]. Regardless of the fundamental differences in purpose and categorization between medical and cosmetic products, drug-free topical medicine is essentially a mixture of ingredients that provide a vehicle/base for the active ingredient, and it is not surprising that much of the cosmetology research overlaps with and is used by pharmacy, particularly in the development and characterization of emulsion systems and the exploration of new ingredients [14].

Over the last five decades, agricultural production has more than tripled due to the expansion of agricultural soil, advances in agricultural technology triggered by the green revolution, and the rapid growth of the world’s population [15]. At the same time, global change driven by consumerist tendencies has led to an alarming acceleration in natural resource extraction and waste generation [16]. Despite the growing interest in biomass conversion technologies, agricultural waste is still underutilized [17]. In many cases, it is simply burned in the fields, especially in developing countries such as Serbia, where there is no legal framework restricting such practices. The burning of crop waste in open fields leads to severe air pollution and raises significant concerns about its impact on the environment and human health, especially among people of all ages, with children being particularly vulnerable to associated health complications [18].

Therefore, the waste generated from the use of natural materials such as fruits, vegetables, grains, and even food is not only a waste of valuable resources in today’s world but also poses a major challenge to the environment [19,20]. By minimizing waste and repurposing discarded products into value-added goods, we can improve the efficiency of our production methods, and develop new green markets and jobs, while simultaneously reaching sustainability goals beneficial for our environment and meeting consumers’ demand [21,22].

Wheat, maize, and sunflower are the most widely grown and harvested crops in the European Union and worldwide [23]. Even though Serbia cannot be compared to the leading producers of crops, it is a fact that its economy is based on agriculture and that maize, wheat, and sunflower produce a total of almost 10 million tons of biomass from crop residues, which often remain completely unused or, even worse, are burned [24]. Although there are numerous methods of waste management, such as waste reduction at source, reuse, recycling, composting, anaerobic digestion, incineration, and other waste treatment processes, the most reasonable and sound approach to solving the problem of organic solid waste, especially crop residues, from a scientific point of view is to investigate the potential of these materials as a free source of various raw materials for the cosmetic, pharmaceutical, and chemical industries [21,25]. Among the abundant sources of agricultural waste, maize, wheat, and sunflower residues stand out for their wide availability and rich biochemical composition.

The formulation of cutaneous products with natural products such as plant extracts represents a multifaceted challenge for the cosmetic and pharmaceutical industries. Plant extracts encompass a vast array of plant-derived compounds, each possessing distinct chemical compositions that significantly influence the efficacy, safety, and shelf-life of cutaneous formulations [26]. In addition to phytochemical variability, the inherent physicochemical properties of plant extracts also pose a formulation challenge, particularly issues such as solubility, compatibility with other ingredients, and susceptibility to degradation [27]. Furthermore, the diversity of plant extracts brings complex formulation issues in achieving the desired sensory properties, texture, and esthetic appearance without compromising product performance, which is considered most important for cosmetics [28,29]. Aside from the technical intricacies, regulatory considerations also complicate formulation challenges associated with botanical extracts, especially safety, efficacy, and labeling.

This research investigates the untapped potential of extracts derived from maize, wheat, and sunflower waste to be used as cosmetic raw materials for cutaneous applications. In order to do this, after the development of the placebo formulation of the emulsion system (i.e., the cream), each extract was incorporated separately into the cream; lipid extracts in 2% by weight and ethanol extracts in 0.1% by weight. For this purpose, the physicochemical and sensory properties of developed creams were investigated using standard methods for characterizing emulsion systems and by conducting an in vivo sensory study. In addition, various in vitro and in vivo studies of creams and pure extracts were performed to investigate the overall potential of these materials for use in cosmetic products and their potential contribution to the structural and functional properties of the topical formulations.

## 2. Materials and Methods

### 2.1. Materials

Mixed emulsifier of 100% natural origin and COSMOS approved, consisting of polyglyceryl-6 distearate, jojoba esters, polyglyceryl-3 beeswax, and cetyl alcohol (Emulium^®^ Mellifera MB, Gattefosse, Saint-Priest, France) was used as a stabilizer in all formulations [30]. The oil phase comprised caprylic-capric triglycerides (Saboderm TCC, Sabo, Levate, Italy) and brassica glycerides (SustOleo™ BG, Inolex, Philadelphia, PA, USA). The water phase was double distilled water preserved with ethylhexylglycerol and phenoxyethanol mixture (Euxyl^®^ PE 9010, Ashland, Düsseldorf, Germany). In creams with lipid extracts, the amount of brassica glycerides was reduced in proportion to the added amount of lipid extract. In order to make the ethanol extracts soluble in the emulsion system, the water phase of the creams with ethanol extracts comprised butylene glycol (Möller Chemie GmbH & Co. KG, Steinfurt, Germany) as a co-solvent, while the amount of water was reduced in proportion to the added amount of ethanol extract.

The harvest residues (stalks and straws) of wheat (*Triticum aestivum*), maize (*Zea mays*), and sunflower (*Helianthus annuus*) were reduced into smaller particles by a grinder and subjected to double extraction as previously described by Glišić et al. [31]. First, they were extracted with hexane at six times their weight (1 h at 40 °C in an industrial stainless steel 60 L extractor). The resulting hexane extracts were vacuum filtered through 87 g/m^2^ filter paper to remove hard residues, and concentrated using a Dlab RE200-Pro rotary evaporator (Dlab Scientific Co., Ltd., Beijing, China) (60 °C, 60 rpm, 216–200 mbar, 150 min). In this way, the lipophilic fraction was isolated and lipid extracts from maize (LE-M), sunflower (LE-S), and wheat (LE-W) were obtained. The residual plant material was left in the open air for 24 h and protected from direct sunlight to allow any remaining solvent to evaporate. The material was then re-extracted for 1 h at 45 °C with 96% ethanol at six times its weight, followed by filtration and evaporation under the same conditions in order to obtain ethanol extracts from maize (EE-M), sunflower (EE-S), and wheat (EE-W). All chemicals—hexane and ethanol—were of analytical grade. The extracts used in this work were previously chemically characterized in terms of their qualitative and quantitative composition by our collaborators on the project and they are presented in Table 1 and Table 2 [32,33,34].

### 2.2. Methods

#### 2.2.1. Development of Cream Formulations Containing Plant Extracts

Cream samples were prepared by heating the water phase in one sealed glass vial and the oil phase with an emulsifier in another glass vial to 70 °C. The oil phase was added to the water phase while stirring at 800 rpm (RZR 2020 laboratory propeller mixer, Heidolph, Scwabach, Germany), and then for 1 min at 1200 rpm. The mixing continued at 800 rpm until room temperature was reached. At temperatures below 50 °C, ethanol extracts dissolved in butylene glycol were added to the creams containing ethanol extracts (WEE, MEE, and SEE). For the creams comprising lipid extracts (WLE, MLE, and SLE), the lipid extracts were added as part of the oil phase. The final formulations are listed in Table 3.

#### 2.2.2. pH and Conductivity Measurements

All pH and conductivity measurements were performed 7 days and 6 months after sample preparation. The pH was measured using the potentiometric method with the calibrated pH meter HI 8417 (Hanna Instruments Inc., Woonsocket, RI, USA). Conductivity measurements were performed with a CDM 230 conductivity meter (Radiometer, Copenhagen, Denmark) calibrated using 0.01 M potassium chloride solution. All measurements were conducted under ambient conditions, in triplicate, and the results were shown as mean ± standard deviation (SD).

#### 2.2.3. Light Microscopy Measurements

An Olympus BX53-P light microscope (Olympus, Tokyo, Japan), coupled with a UC50 CCD color camera (5 MPa) and cellSens 1.15 Life Science Imaging Software, was used to evaluate the microstructure of the examined samples at 400× magnification. Also, the diameter of 500 droplets was measured for each sample using the ImageJ software, version 1.52a (National Institutes of Health, Bethesda, MD, USA). The mean droplet diameter and SD were calculated (minimum and maximum measured diameters are also shown).

#### 2.2.4. Differential Scanning Calorimetry (DSC) Measurements

All samples were accurately weighed in an amount of 5–10 mg and then crimped into standard aluminum pans (40 μL) with a perforated lid. The samples were heated from 25 to 120 °C (heating rate 10 °C/min) under a constant nitrogen flow (50 /mL/min) on the DSC 1 instrument (Mettler Toledo, Greifensee, Switzerland), coupled with STAR^e^ software version 12.10. The empty sealed pan was used as a reference.

#### 2.2.5. Rheological Measurements

Continual rheological measurements on creams were conducted in triplicate for 7 days and 6 months (stored at room temperature) after their preparation using a rotational rheometer (Rheolab MC 120, Paar Physica, Stuttgart, Germany). The measurements utilized a cone/plate measuring system (with a diameter of 50 mm and an angle of 1°), employing a sample thickness of 0.05 mm and maintaining a temperature of 20 ± 0.1 °C. A controlled shear rate procedure was employed, ranging from 0 to 200 s^−1^ and back to the initial point, with each phase lasting 120 s.

#### 2.2.6. In Vitro Assessment

##### Texture Analysis

Texture analysis of investigated creams was performed using an EZ-LX Texture analyzer (Shimadzu, Kyoto, Japan) at room temperature, with a compression test. The method consists of immersing the probe (in this case a cup-like extension was used) into the cream sample (75% of the packaging is filled with the cream sample) at a predefined speed (2 mm/s) and a predefined distance (up to 1 mm) from the bottom of the packaging. All measurements were performed in triplicate at a constant temperature of 20 ± 2 °C. The results obtained using the integrated software package Trapezium-X Single v1.5.2 are shown as the mean of three repeated measurements ± SD.

##### Spreadability

The spreadability was evaluated according to Djekic et al. [35]. An accurately measured amount (0.1 g) of the sample was placed on a flat glass surface (inside a circle with a diameter of 1 cm) and covered with another glass plate. Then, the sample was subjected to a mass load of 50 g under ambient conditions. After 5 min, the largest diameter of the sample after spreading was measured, and the spreadability coefficient (Φ) was determined. The results are expressed as the mean of 10 repeated measurements ± SD.

##### Skin Occlusivity

To evaluate the occlusive properties of creams, an in vitro occlusion test was performed [36]. Beakers (100 mL, diameter 4.9 cm) were filled with 50 mL of purified water, and covered with filter paper, and 250 mg of the sample was evenly distributed on the upper surface of the filter paper. The beakers were subsequently stored at 32 ± 0.5 °C for 48 h in order to mimic the temperature of the skin surface. The beaker with water covered only with the filter paper was used for calculations and the beaker with water covered with filter paper and 250 mg of white soft paraffin/petrolatum was used as a reference for maximal occlusion factor. The evaporation of water was measured and the occlusion factor “F” was calculated at 24 and 48 h following Equation (1), where A represents the water loss without the sample and B represents the water loss with the sample. An F = 0 means no occlusive effect compared to the reference, while an F = 100 means maximum occlusivity.

F = [(A − B)/A] × 100(1)

##### Water Washability

Water washability was evaluated by applying the cream formulation in the amount of 0.05 g on a microscope slide or PVC oilcloth attached to a microscope slide, both of known mass (approximately 2 mg/cm^2^ of the material), and spreading it to leave a thin layer. The applied sample was left for 5 min to dry, and then the mass was measured again. Then the slide with the dried sample was washed (not under a strong stream) with 50 mL of purified water, the excess water was gently absorbed, and the slide was left to dry in the oven at 40 °C until constant mass. After drying, the mass was measured again. The water washability was expressed as a percentage relative to the amount of sample immediately before washing, as the mean of three repeated measurements ± SD.

##### Stickiness

The stickiness was evaluated according to Umar et al. with some modifications [37]. Subsequently, 0.05 g of the cream formulation was applied to a microscope slide or PVC oilcloth attached to the microscope slide, both of known mass (approximately 2 mg/cm^2^ of the material), and spread to leave a thin layer. The applied sample was left for 5 min to dry, and then the mass was measured again. Cotton wool was applied to the prepared sample with gentle pressure. The amount of sample remaining on the cotton wool after gentle pressure was calculated and the results are expressed as a percentage relative to the amount of sample on the slide before pressing with cotton wool (100%), as the mean of three repeated measurements ± SD.

##### Cytotoxicity MTT Assay

The effect of extracts on the cell survival of normal human keratinocytes (HaCaT) was determined using the MTT assay. Stock solutions of extracts were prepared in dimethyl sulfoxide (DMSO) at the concentration of 600 µg/mL and then diluted to the appropriate working concentration. The HaCaT cell line (obtained from American Type Culture Collection, Manassas, VA, USA) was cultured in complete culture medium RPMI-1640 (Sigma Aldrich, St. Louis, MO, USA) at 37 °C in a humidified atmosphere with 5% CO_2_. HaCat cells (7 × 10^3^ cells/100 µL per well) were added in triplicate to flat-bottomed 96-well microtiter plates. After 20 h, samples were added at five different concentrations ranging from 12.5 μg/mL to 200 μg/mL. Only the culture medium was added to the cells in the control wells (e.g., positive control). All experiments were performed in triplicate.

The MTT assay was performed for cell survival determination after 48 h of incubation by measuring the absorbance of the cell medium at 490 nm. For cell survival determination, 3-(4,5-dimethylthiazol-2-yl)-2,5-diphenyltetrazolium bromide—MTT (Sigma Aldrich, USA) was dissolved in phosphate-buffered saline (PBS) and added to each well. After 48 h of incubation and an additional 4 h at 37 °C, 5% CO_2_ in a humidified atmosphere, 100 µL of 10% sodium dodecyl sulfate (SDS, quality level 100, Sigma Aldrich, USA) were added to each well and the absorbance was measured at 490 nm the next day. The measurements were performed using a Multiskan^®^ FC Microplate Photometer (Thermo Scientific, Waltham, MA, USA). The cell survival rate (%) was calculated as follows: The absorbance at 490 nm of the samples containing cells grown in the presence of the test compounds was divided by the absorbance of the positive control sample after subtracting the absorbance of the blank sample. The IC_50_ values were determined by numerical analysis of the data obtained from the graph as the concentration of the compound that reduced the survival of the treated cells by 50%.

##### Antimicrobial Activity

The antimicrobial activity of the extracts and creams was tested against ATCC laboratory control strains of seven pathogenic bacteria and one yeast purchased from the ATCC and NCIMB collection (KWIK-STIK™, Microbiologics, St. Cloud, MN, USA) as follows: *Staphylococcus aureus* subsp. *aureus* Rosenbach ATCC 6538, *Staphylococcus epidermidis* ATCC 12228, *Enterococcus faecalis* ATCC 29212, *Escherichia coli* ATCC 25922, *Klebsiella pneumoniae* subsp. *pneumoniae* NCIMB 8267, *Salmonella enterica* subsp. *enterica* serovar Abony NCTC 601, *Pseudomonas aeruginosa* ATCC 27853, and *Candida albicans* ATCC 24433. The strains were maintained at −80 °C in 15% glycerol, defrosted before experiments, and inoculated from stock solutions onto Triptic Soy agar/broth (TSA/TSB, Oxoid Ltd., Basingstoke, UK) for bacteria and Sabouraud Dextrose agar/broth (SDA/SDB, Oxoid) for *Candida albicans*. Prior to experiments, all tested strains were incubated on corresponding agar plates for 24–48 h at 35 °C under aerobic conditions, and fresh (overnight) cultures were used for preparation of inoculum.

The antimicrobial activity of extracts was determined by broth microdilution test in 96-well microtiter plates according to the European Committee for Antimicrobial Susceptibility Testing guidelines [38,39]. The suspensions of microorganisms were made in a saline solution to a density of 0.5 per McFarland standard (bioMérieux, Craponne, France). Extracts were dissolved in DMSO, and further prepared in concentrations ranging from 64–0.125 mg/mL in fresh Mueller-Hinton broth (MHB, Lab M Limited, Bury, UK) for bacteria and SDB for yeast. Each concentration was set in duplicate and inoculated with 5 × 10^5^ CFU/mL of microorganisms. For the detection of cell growth and metabolism, MHB/SDB were supplemented with 0.05% 2,3,5-triphenyltetrazolium chloride (TTC, Sigma-Aldrich-Merck KGaA, Darmstadt, Germany). TTC is a redox indicator and a colorless dye that becomes a red metabolite 1,3,5-triphenyformazan due to the activity of cellular dehydrogenase and is used to differentiate living cells from dead cells. Minimal inhibitory concentrations (MIC) were determined after incubation for 20–48 h at 35 °C in aerobic conditions as the lowest concentration of extract that inhibits growth (i.e., shows no visible change of medium color). Positive controls (microorganisms in medium) and negative controls (only medium with extracts) were included in the experiments. Each test was repeated three times.

#### 2.2.7. In Vivo Assessment

##### Sensory Analysis

Fifty females without extensive training aged between 23 and 49 years (average age 27.26 ± 6.36 years) were recruited for sensory evaluation. Assessors signed an informed consent form approved by the Local Ethics Committee of the University of Belgrade, Faculty of Pharmacy (No. 761/2) in accordance with the Declaration of Helsinki. Prior to the assessment, volunteers rested in acclimatized premises (temperature 22 ± 1 °C and relative humidity 40 ± 5%) for 15 min.

The formulations were marked with three-digit codes in jars, and certain attributes were evaluated by looking and smelling. When necessary, in order to investigate attributes related to rub-in and after-feel phase, similar amounts of formulation (2 mg/cm^2^) were applied on an upper area of the palm using an applicator. Volunteers answered a CATA (Check-All-That-Apply) questionnaire related to five domains of product: product look in the jar, product color, skin feel in the rub-in phase, skin feel in the after-feel phase, and product smell. The panelists were presented with a list of attributes/sentences and asked to check each of the offered terms/answers that they considered appropriate to describe their sensations related to the investigated sample [40].

##### Efficacy

In vivo assessment was performed for samples with lipid extracts in a 24-h short-term study by non-invasive biophysical measurements of the skin. The panel of 20 healthy volunteers (age 34 ± 8 years) participated, after signing a written consent form (No. 761/2) in accordance with the Declaration of Helsinki. Two test sites on one arm and three test sites on the other arm (flexor aspects of forearms) were used to test four samples and one was used as non-treated control (NC). The application scheme was randomized in advance and the measuring investigator was not acquainted with the scheme. In the short-term 24-h study, stratum corneum moisturization (SCM) was measured before and 1 and 24 h after application of samples, while transepidermal water loss (TEWL) was measured prior to sample application and 24 h after.

All measurements were conducted under controlled temperature (22 ± 1 °C) and humidity (40 ± 5%) conditions after a 30 min acclimatization period. Skin biophysical characteristics were measured according to relevant guidelines [41,42]. A calibrated Cutometer^®^ MPA580 (with integrated Corneometer^®^CM825) and Tewameter^®^ TM210 (all Courage + Khazaka, Köln, Germany) were used for the measurement of skin SCM and TEWL.

##### Statistical Analysis

Sensorial data were analyzed to compare the effects of different treatments using Cochran’s Q test and Chi-squared test for contingency tables. The analyses were performed using Python 3.9 in the Google Colab environment. Cochran’s Q test assessed significant differences in binary response variables across multiple products, while pairwise comparisons between products used Chi-squared or Fisher’s exact test with Bonferroni correction for multiple testing and McNemar’s test for every pair of answers within the sample. Results with *p*-values < 0.05 were considered significant.

For comparison of raw data of in vivo study, one-way ANOVA parametric analysis with post hoc Tukey’s test was used, and for nonparametric distribution, the Kruskal-Wallis with Dunns post test was conducted (SigmaStat4.0) and significance levels were *p* < 0.05.

For all other results expressed as means ± standard deviations, when applicable, *t*-test (*p* < 0.05) was employed for all analysis. For skin occlusivity, two-way ANOVA was performed with post hoc Bonferroni *t*-tests where appropriate for all pairwise comparisons within the data set.

## 3. Results and Discussion

### 3.1. Formulation Development

With the aim of investigating the potential of extracts from harvest residues to be used as cosmetic ingredients in cutaneous formulations, we set out to develop a simple cream (placebo 1 formulation—P1) that would also be in line with sustainable development in terms of ingredients. Initially, we prepared formulations with different concentrations of extracts—lipid extract 1.0, 2.0, and 10.0% and ethanol extract 1.0, 0.5, and 0.1%. As the formulations were only allowed to differ in the type of extract, due to the instability of the cream with 10% of the maize lipid extract, we selected formulations with 2% lipid extract for further investigation—WLE (wheat lipid extract in P1), MLE (maize lipid extract in P1), and SLE (sunflower lipid extract in P1). In all formulations with ethanol extracts, butylene glycol was added as a necessary co-solvent and placebo 3 (P3) was prepared. All formulations with 1% ethanol extracts were not stable few days after preparation as well as the formulation with 0.5% sunflower ethanol extract, so formulations with 0.1% ethanol extract were selected—WEE (wheat ethanol extract in P3), MEE (maize ethanol extract in P3), and SEE (sunflower ethanol extract in P3). The detailed composition of the selected formulations is given in Table 3.

The initial development of formulations has shown that the extracts studied can be incorporated into formulation in larger quantities than in the creams tested. Lipid extracts can be incorporated in concentrations of up to 10%, whereas this concentration is much lower for ethanol extracts and could be as low as 0.5% according to our results. However, to achieve this, case-by-case development of each individual formulation is required.

### 3.2. pH and Conductivity

The addition of lipid and ethanol extracts to the placebo formulations resulted in a small change in pH (Table 4). Sunflower lipid (SE-L) and ethanol (SE-E) extracts had the greatest effect on lowering pH compared to their respective counterparts. As pH is one of the parameters monitored in cosmetics stability test protocol in order to assess the stability of semi-solid preparations, its values after 6 months gave us an insight into the preliminary stability of the developed formulations [43]. After 6 months of storage at room temperature, a slight decrease was observed in the formulations with lipid extracts and an increase in the formulations with ethanol extracts. However, initially, all values were in the range of 6.49–7.35, and after 6 months in the range of 6.65–7.35. The changes in the placebo formulations corresponded to those in the formulations with extracts. Although the changes in pH values after 6 months for all samples except WLE and SLE were statistically significantly compared to the values after 7 days, the more appropriate assessment for this parameter is whether this parameter remained within the 90% confidence interval [44]. pH remained within the acceptance criteria of ±10%.

Similar to the pH value, the conductivity of the placebo formulations also increased with the addition of extracts, although it was more pronounced with ethanol extracts (Table 4). Conductivity measurements are carried out in order to define emulsion type and can be used as a reliable and fast method to detect changes in emulsion structure over time [45]. Although statistical analysis showed significant changes for all samples except P1, SLE, and P3 in the course of 6 months, the obtained results could be considered satisfactory since there were no changes in emulsion type or an extreme change in conductivity.

Overall, the measurements of pH and conductivity at different time points indicate good preliminary stability of investigated samples. The fact that the obtained differences are statistically significant indicates that in the commercialization and development of complex formulations, more attention should be given to the stabilization of these products. The changes in pH values were highest for wheat extracts, then for maize, and the lowest for sunflower extract. It is interesting to note that the highest total phenolic and flavonoid content was in the ethanol extract of wheat, followed by maize, and sunflower (Table 1). These results suggest that for formulations with high phenolic and flavonoid extracts, better, additional stabilization, e.g., through the use of antioxidants or rheology modifiers, is required. Although the emulsifier is an oil-in-water type, conductivity values below 50 µS/cm indicate a specific stabilization mechanism of an emulsifier and water being trapped within the lamellar phase and unavailable for conductivity measurements [46,47]. The fact that lipid extracts as components of the oil phase induce changes in conductivity could mean that their presence interferes with these specific structures. Moreover, certain components of the lipid extract could be solubilized, which is in accordance with the data showing that the interaction of surfactant (emulsifier) with fatty acids affects their solubilization [48]. As expected, ethanol extracts led to a greater increase in the conductivity of the placebo formulation when added to the water phase, which has been shown to be the outer phase of creams. Based on the composition of ethanol extracts (Table 1), the largest number of isolated compounds (*p*-coumaric acid, ferulic acid, and tricin) was in the maize ethanol extract, which could explain the highest conductivity values of the MEE sample.

### 3.3. Light Microscopy

The results in Table 5 show the dimensions of the emulsion droplets in the creams tested. After the addition of lipid extracts, the distribution and mean droplet size of the emulsion were smaller compared to the placebo (P1). In the samples with ethanol extracts, a slight decrease in mean droplet size and a significant decrease in droplet size distribution were observed compared to P3, with the exception of SEE.

Light microscopy showed that the addition of extracts led to a reduction in droplet size and a more homogeneous distribution of droplet size, indicating better stability, except for the SEE sample [49].

### 3.4. Differential Scanning Calorimetry (DSC)

**Figure 1:**
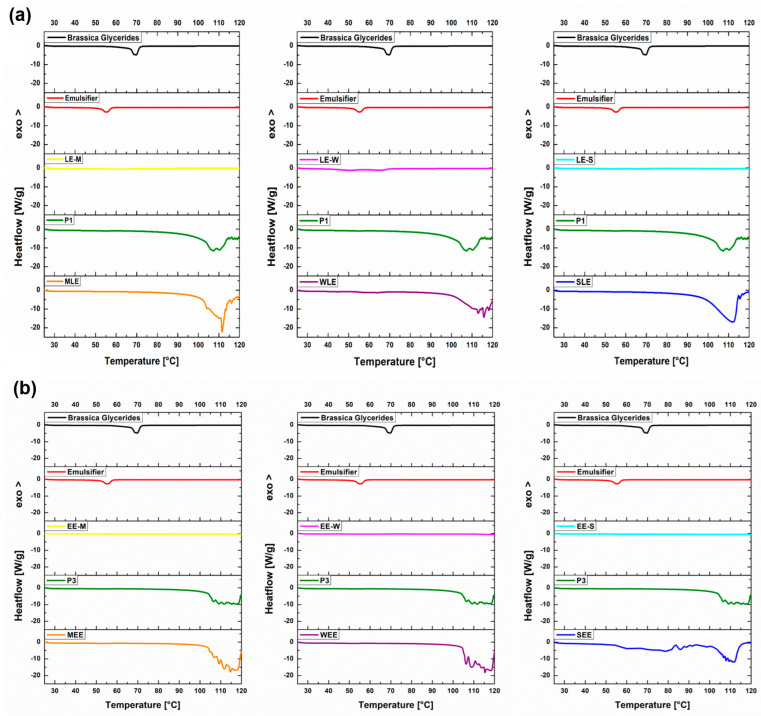
DSC thermograms of (**a**) creams with lipid extracts and (**b**) creams with ethanol extracts—thermograms of solid lipid cream ingredients (brassica glycerides and emulsifier), pure extract, placebo cream, and cream with extract are presented for each sample.

DSC analysis was carried out in order to evaluate the effects of extract addition on the structure of the placebo formulations. The samples were submitted to steady heating in order to evaluate their thermal behavior. For this reason, the DCS thermograms in Figure 1 are plotted so that each graph shows the thermal behavior of the solid lipids (brassica glycerides and emulsifier) contained in each formulation, as well as the respective extract itself, the placebo formulation of the cream, and the corresponding cream with the extract. The DSC thermograms showed single endothermic peaks on the curves of solid lipids (for brassica glycerides at 70 °C and the emulsifier at 56 °C). These peaks are the result of the melting of the mentioned ingredients and indicate that the pure solid lipids have transitioned to the liquid state and then to an anisotropic liquid crystalline phase [50]. Similar melting temperatures were observed for the pure lipid extracts (63 °C for LE-M, 58 °C for LE-W, and 60 °C for LE-S), although this is not visible in the thermograms, due to the very low enthalpies. These lower melting enthalpies of the lipid extracts compared to the brassica glycerides could be due to the high concentration of free fatty acids and the presence of 13.02–21.68% of other components in lipid extracts that are not fatty acids and triglycerides [33,34]. When water and oil were added to solid lipids and creams were made, transitions observed in brassica glycerides and emulsifiers were not seen. For placebo creams P1 and P3, a single broad endothermic peak is observed, starting at about 95 °C and centered around 110 °C, which corresponds to water evaporation [51]. The shift of the peak center to temperatures above 100–105 °C, which is characteristic of the evaporation of water itself [52], indicates the formation of structures that stabilize complex emulsion systems in which water remains entrapped as interlamellarly fixed, such as in placebo creams P1 and P3 [53]. Creams with lipid extracts are characterized by a similar broad endothermic peak. The addition of lipid extracts resulted in a slight broadening of the peaks and a slight shift of their centers towards higher melting temperatures compared to P1 (116 °C for WLE and 113 °C for MLE and SLE). Although the focus of this work was not on the emulsifier stabilization mechanism, the obtained results imply the formation of specific structures in creams that require higher temperatures for phase transitions [46,54,55]. When 2% of brassica glycerides were altered with lipid extracts (in WLE, MLE, and SLE), there were no drastic changes in the thermal behavior of the creams compared to P1. This could be explained by the similar fatty acid profile of brassica glycerides consisting of palmitic (C16:0), stearic (C18:0), oleic (C18:1), linoleic (C18:2), linolenic (C18:3), and erucic acid (C22:1) and our lipid extracts [56]. Romanić et al. confirmed that the most common constituents in lipid extracts were fatty acids—unsaturated fatty acids, mainly consisting of linoleic acid (C18:2) and oleic acid (C18:1), polyunsaturated fatty acids, alpha-linolenic acid (C18:3), and among the saturated fatty acids, palmitic acid was predominant—while only 21.68%, 13.02%, and 16.85% of the lipid extracts from wheat, maize, and sunflower harvest residues, respectively, were other components [33,34].

The main differences between creams with different lipid extracts were the significantly higher water evaporation enthalpies (985.22 J/g for MLE, 824.73 J/g for WLE, and 1153.23 J/g for SLE) compared to the placebo formulation P1 (523.37 J/g), which correlated with the results of the conductivity measurements of these creams. The addition of extracts, particularly LE-M and LE-S, yields creams in which the water phase is an outer phase (MLE and SLE had an initial conductivity above 50 µS/cm) and therefore evaporates more readily from the system. No peaks were observed on the thermograms of the ethanol extracts (EE-M, EE-W, and EE-S) at temperatures from 25 to 120 °C, suggesting that these extracts are not subject to thermal changes in the indicated temperature range, which is to be expected given their polyphenolic nature [32]. The DSC thermograms of the creams with ethanol extracts (which were dissolved in the aqueous phase) were very similar to those of the placebo (P3). Only the cream with sunflower ethanol extract (SEE), showed an endothermic peak at 55–90 °C, which could be associated with earlier water evaporation, i.e., evaporation of water from the outer phase of the emulsion, which could also indicate a potentially lower stability of this cream compared to the others. In this sense, the DSC results can be related to the results of the droplet size measurements (the largest droplet size distribution of SEE), although other characterization methods showed satisfactory stability of all samples after 6 months. Previously, we have not been able to formulate creams with 0.5% ethanol extracts because the sample with EE-S was inhomogeneous immediately after preparation. Considering the aim of our work to investigate the possibility of developing topical formulations, these results show that formulations with ethanol sunflower extract should be given more attention compared to those with wheat and maize ethanol extracts.

### 3.5. Rheology

**Figure 2:**
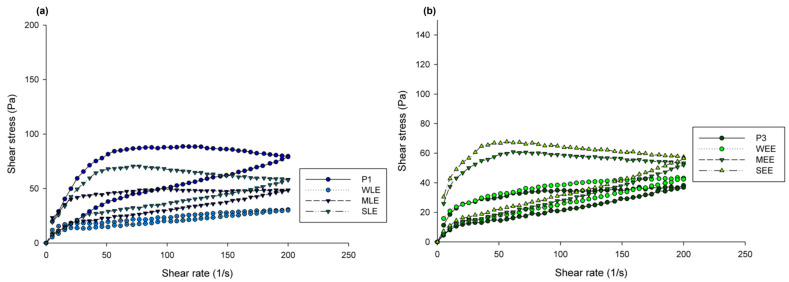
Shear stress vs. shear rate curves of (**a**) P1 and creams with lipid extracts and (**b**) P3 and creams with ethanol extracts.

Continuous rheological measurements were performed in order to investigate the influence of added extracts on cream structure together with certain sensory characteristics important for consumers’ satisfaction with topical products. In Figure 2, initial rheograms of creams show that all samples exhibited thixotropic behavior. The addition of lipid extracts, with the exception of wheat extract, resulted in a lowering of shear stress at corresponding shear rates compared to P1, while the opposite happened upon the addition of ethanol extracts to the P3 formulation. Changes in flow behavior and viscosity after 6 months of storage at room temperature compared to initial measurements showed that within all samples, certain thickening occurred but without significant change in overall rheological behavior. In Table 6, apparent maximum and minimum viscosities of investigated samples initially and after 6 months are presented.

Thixotropic behavior, which indicates shear thinning of the products under external force, is desirable for topical products as it allows easy spreading and slipping of the product. Our results show that the changes that occurred when altering brassica glycerides with lipid extracts from wheat, maize, and sunflower were not pronounced. As we have already discussed in relation to thermal behavior, it is evident that there is good co-stabilization between the emulsifier used and the brassica glycerides, and rheological measurements, consistent with DSC measurements, show that the lipid fraction from harvested material could also be used as a structural ingredient in creams as a component of the lipid phase. It should be mentioned that with higher concentrations of ethanol extracts (0.5 and 1%), it was not possible to formulate stable creams. Similar to lipid extracts, the addition of a small amount of ethanol extracts had no significant effect on the flow behavior. The addition of ethanol extracts of sunflower and maize resulted in a slight thickening and of the wheat extract in the thinning of the placebo cream. Overall, rheological measurements indicate that the addition of plant residue extracts did not disturb the existing structure of the creams.

Repeated rheological measurements (after 6 months of storage at room temperature) together with visual assessment (color, smell, and homogeneity), pH, and conductivity measurements indicate satisfactory physical stability of investigated samples. Based on the obtained results, in regard to the correlation between rheological flow properties and certain sensory attributes, it can be assumed that the application of all investigated topical products (mainly spreading and slipperiness) would be similar [29].

### 3.6. In Vitro Assessment Analysis

Texture analysis enables the characterization of samples based on their mechanical properties. The applied method is used for semisolid preparations, and parameters obtained in this way are firmness (maximum on the positive curve), consistency (area under the positive curve), cohesiveness (maximum on the negative curve), and index of viscosity (area under the negative curve) [57]. The texture parameters of investigated creams are shown in Supplementary Material in Table S1. The firmest sample is P1, followed by SLE, SEE, MLE, MEE, WEE, WLE, and P3, which stands in good agreement with the shear stress values of investigated samples when compared at the same shear rate. Although the results of the texture analysis are consistent with the rheological measurements, it should be noted that the extracts did not lead to significant changes in the mechanical properties of the creams. This is particularly important as plant ingredients can be problematic in terms of product formulation and especially sensory application properties, which is the reason for consumer dissatisfaction. This is often the reason why consumers who want healthier and more sustainable products with green ingredients no longer buy those after their first purchase [58]. These results imply similar sensory properties related to the application and rub-in phase of the placebo and the formulations with extracts.

**Figure 3:**
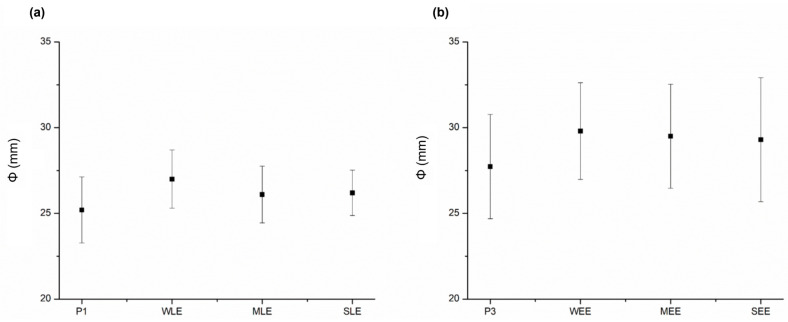
Spreadability coefficient—Φ (mm) of investigated creams: (**a**) P1 and creams with lipid extracts, (**b**) P3 and creams with ethanol extracts.

Spreadability was assessed using a simple technique, which could be a reliable method to compare spreadability in the case of very similar formulations (Figure 3). Sample P3 and the corresponding samples of this placebo with ethanol extracts showed slightly higher spreadability values compared to P1 and the creams with lipid extracts, consistent with flow curves (lower shear stress values for corresponding shear rates). The WEE and WLE samples showed the highest spreadability among the samples with ethanol and lipid extracts, respectively. The in vitro evaluation of spreadability showed small differences between the samples, without statistical significance, which is in accordance with rheological and textural measurements [29].

**Figure 4:**
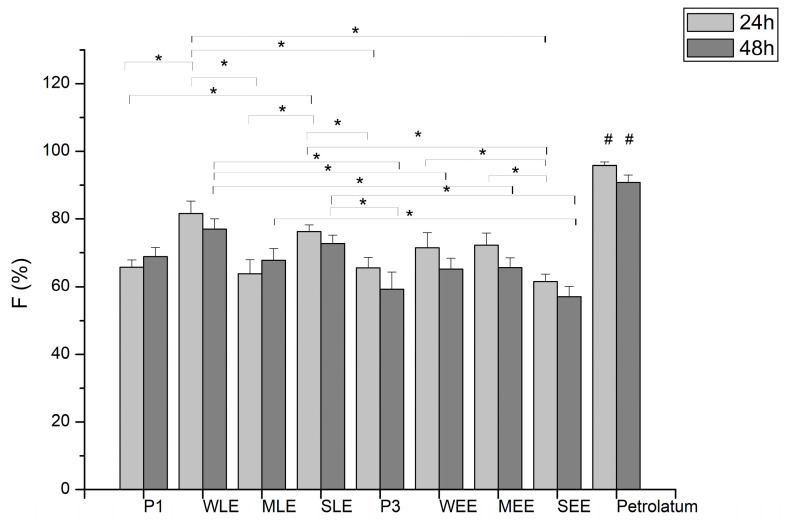
Occlusion factors (F) of creams obtained by in vitro occlusivity test; statistically significant changes between investigated samples are labeled with * and # stands for statistically significant changes between petrolatum and all investigated samples at different time points.

In addition to spreadability, another in vitro method can be used as a reliable method to evaluate the behavior of topical formulations during application (rub-in phase), but more importantly to predict the efficacy of emollient products. The skin occlusivity test was performed to calculate the occlusion factor (F). Based on the occlusion factor obtained, lipid extracts from wheat and sunflower increased the occlusivity of P1, while MLE and P1 had similar effects (Figure 4). A statistically significant increase for WLE and SLE samples compared to the placebo formulation P1 was obtained only after 24 h, while an increase after 48 h is not significant except in comparison with P3 and some samples with ethanol extracts. Ethanol extracts from wheat and maize slightly increased the occlusivity of P3, and the SEE sample had a slightly lower occlusivity than P3. After 24 h, the MEE and WEE samples had a significantly higher occlusivity compared to SEE, while after 48 h, there was no statistically significant difference between the ethanol samples.

However, skin occlusivity was not related to any previous results. Occlusives are considered a class of moisturizers that physically block transepidermal water loss (TEWL) in the stratum corneum (SC), and this method mimics the occlusive effect at a certain level [59]. Based on this fact and our results, it can be assumed that creams with higher F-values have a more pronounced occlusive effect on the skin, but also that their spreading properties might be better. There is a good agreement between the spreadability and occlusion tests, as the order of F-values after 48 h and spreadability is the same for all tested samples with lipid extracts, while for samples with ethanol extracts, it is the same for WEE and MEE. Another class of moisturizers are emollients—mainly lipids and oils that soften and smoothen the skin and improve its elasticity and moisturizing ability; they cannot be strictly distinguished from occlusives [59]. The topical application of emollients creates a thin layer on the skin surface that prevents water evaporation and improves skin moisturization [60,61]. In vitro efficacy results indicate that the investigated extracts, especially lipid ones, contribute to the emollient properties of the topical formulations.

The assessment of stickiness and water washability was only performed for P3 and samples with ethanol extracts and both procedures were performed on glass plate and PVC oilcloth (Figure S1). The results for stickiness showed a good correlation between glass and PVC substrates used, while the results for washability could not be correlated. WEE differed from other samples and showed the highest values for stickiness and the lowest values for washability on glass. The results obtained for washability and stickiness can be used to compare formulations, but only within a defined substrate. If we discuss the results on glass, the stickiest sample was WEE, the P3 sample was the one with the highest washability, and the WEE was the one with the lowest washability. These reciprocal results make sense, as it can be assumed that the stickiest product is the most difficult to rinse off. From all the results, we can only conclude that the addition of a small amount (0.1%) of the ethanol extract affects the washability and stickiness of the initial formulation P3. However, it is not possible to interpret the results obtained in terms of in vivo properties, i.e., the behavior of the product on the skin. Furthermore, our results show that additional tests should be performed for these in vitro tests in order to establish correlations between them.

#### 3.6.1. Cytotoxic MTT Assay

**Figure 5:**
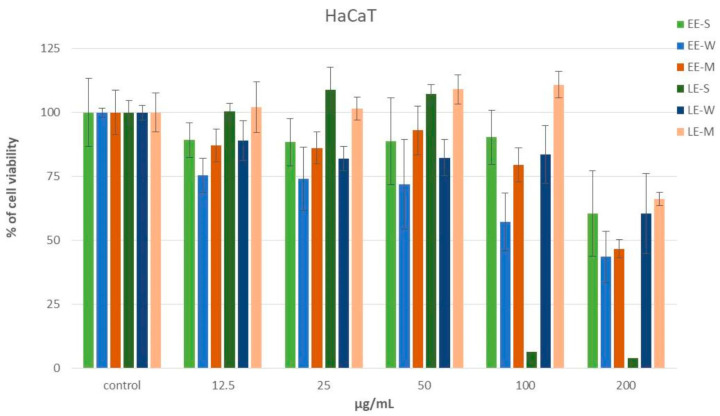
The effect of investigated extracts on cell viability of human keratinocyte cell line HaCaT.

To be used, any new ingredient must meet high requirements, especially in terms of its safety. Medical skincare products are subject to extremely restrictive requirements, but according to EU regulations, safety assessment is also mandatory for cosmetic products and is based on the safety assessment of each ingredient of the cosmetic product [5]. Since animal testing is not allowed for this purpose, alternative in vitro methods are used and MTT assay is used as a standard to study cell toxicity and for screening formulations and ingredients safety [62]. In this assay, if the cell viability is less than or equal to 50%, the tested sample is potentially unsafe and may be treated as a local irritant [63]. To evaluate the safety of materials from harvest residues, lipid and ethanol extracts were investigated using the human keratinocyte cell line HaCaT, and cell viability was assessed. Cells were treated with sample concentrations of 12.5–200 μg/mL and none of the extracts studied at concentrations of 12.5–50 μg/mL reached the threshold of 50% cell viability. Proliferation of HaCaT cells ranged from 71.9% to 109.1% compared to the positive control (Figure 5). The IC_50_ was higher than 200 μg/mL for EE-S, LE-W, and LE-M extract, 190 μg/mL for EE-M, 150 μg/mL for EE-W, and 80 μg/mL for LE-S. The results obtained show that maize lipid extract (LE-M) had the best effect on cell proliferation in the concentration range studied, while the good cell proliferating effect of sunflower lipid extract (LE-S) at lower concentrations was replaced by a cytotoxic effect at concentrations above 80 µg/mL. For all extracts, a certain linear concentration–effect relationship is evident starting at 50 µg/mL, suggesting that extracts at higher concentrations might have a cytotoxic effect on human keratinocyte cell lines.

The cytotoxic MTT test showed a good safety profile of all extracts at concentrations of 12.5–150 μg/mL, except for the sunflower lipid extract (IC_50_ higher than 80 µg/mL). We consider these results satisfactory considering that the extracts were investigated in their pure form. The good effect on cell proliferation for maize lipid extract at all concentrations studied and for sunflower lipid extract at lower concentrations could be relevant for the use of these extracts as active ingredients. The decrease in cell proliferation with increasing extract concentration indicates that the concentration of the extract should be carefully considered during formulation. Nevertheless, it can be assumed that the extracts in the tested samples with low extract concentrations are not potentially irritating to the skin and that a satisfactory safety profile of formulations with these ingredients can be achieved in combination with other safe ingredients.

#### 3.6.2. Antimicrobial Activity Analysis

Preservatives, as ingredients that prevent the growth and development of microorganisms, are necessary in water-based topical products [63]. On the other hand, due to the possible toxic effect of preservatives, there is a requirement that products should be free of preservatives. For this reason, research into multifunctional ingredients that could also have antimicrobial effects is attracting great attention [64]. We investigated the antimicrobial activity of all the extracts tested, not only on those microorganisms whose presence is not allowed in cosmetic products and non-sterile topical medicines but also on several microbial laboratory strains that could be of importance for the human microbiome [65].

**Figure 6:**
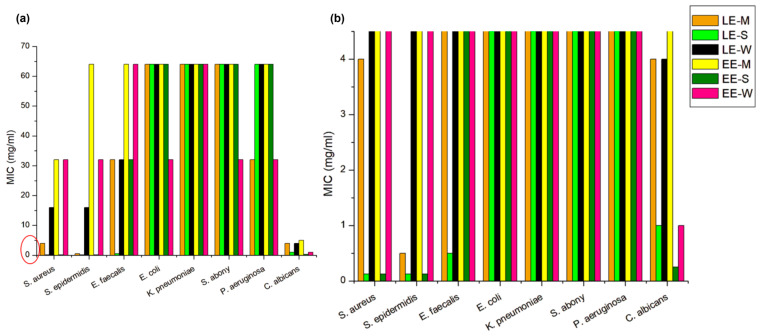
Antimicrobial activity of investigated extracts (**a**) MIC 0–64 mg/mL and (**b**) MIC 0–4.5 mg/mL (the part of the graph marked with a red circle in Figure 6a enlarged for a better view).

The results of the antimicrobial activity of all extracts are presented in Figure 6, with MIC values ranging from 0.125 to 64 mg/mL. As expected, the preserved cream samples showed low antimicrobial activity against all bacteria and yeasts tested with a MIC value of 64 mg/mL or more. Sunflower ethanol extract (EE-S) showed the best activity against *S. aureus* and *S. epidermidis* (MIC 0.125 mg/mL and less). Wheat ethanol extract (EE-W) showed moderate activity against these strains (MIC 32 mg/mL) and maize ethanol extract (EE-M) showed moderate and low activity against *S. aureus* and *S. epidermidis* (MIC 32 and 64 mg/mL, respectively). All ethanol extracts showed the best activity among investigated microorganisms (MIC < 1 mg/mL) against *C. albicans*. All lipid extracts showed good antimicrobial activity against *S. aureus*, *S. epidermidis*, *E. faecalis,* and *C. albicans*, while maize lipid extract (LE-M) showed low antimicrobial activity against *P. aeruginosa* (MIC 32 mg/mL). LE-S showed the best antimicrobial activity among lipid extracts.

The results of the antimicrobial activity of the ethanol extracts were expected due to their composition and overall flavonoid and phenolic compounds that exhibit antimicrobial activity [32,66]. The more pronounced effect of sunflower extract could be explained by the presence of chlorogenic acid, which has been shown to have a wide range of antimicrobial activity [67]. A slightly better effect of wheat extract compared to maize extract may be due to the higher content of coumaric acid [68]. The fact that the lipid fraction also showed very good antimicrobial activity is more interesting. The antimicrobial properties of fatty acids (FA) have long been recognized, and for saturated FA, better antimicrobial activity is determined for FA with a shorter alkyl, while unsaturated FA with a long chain has a better effect than saturated ones [69,70]. It has been shown that a moderate alkyl chain length and the presence of an unsaturated site C8:0–18:1 FA are required for antimicrobial activity and this could explain the antimicrobial activity of the extracts studied [71]. Based on available data for unsaturated FA antimicrobial activity, it could be assumed that LE-S (lipid sunflower extract) showed the best antimicrobial activity due to the highest content of unsaturated FA—C18:1, C18:2, and C18:3 (Table 2: LE-S ~ 61%, LE-M ~ 56%, and LE-W ~ 41%)—among lipid extracts. Considering the great interest in the research of FA antimicrobial effect and their application in the field of food, medicine, and cosmetics, it is important to emphasize the antimicrobial activity of investigated lipid extracts from harvest waste, which as structural components of an oil phase in an emulsion could be considered as multifunctional ingredients of topical products [72,73]. Nevertheless, as in the case of tested creams with extracts that did not show antimicrobial activity, the fact that extracts have an antimicrobial effect is not enough to assume such activity in the final formulation, and this potential effect needs to be evaluated for each formulation separately. In addition, this is important due to the fact that extracts can have a negative impact on skin commensal bacteria such as *S. epidermidis*. For potential final formulations in which extracts would be a part of the preservative system, proper safety assessment regarding the effect on the skin microbiome should be performed.

### 3.7. In Vivo Assessment Analysis

Although our main goal was to successfully formulate stable products for topical application using materials derived from harvest residues, we quickly realized that these materials strongly influence the sensory properties of the product—primarily the appearance, i.e., the color and smell of the product. Since sensory properties are essential from a cosmetic perspective and are undoubtedly important for the adherence and compliance of topical medications, we conducted a sensory study using a Check-All-That-Apply (CATA) questionnaire.

For the first investigated domain ‘look in a jar’ (Figure S2), attributes thick/creamy, colored/not white, glossy, matt/not glossy, thin/milky, grainy, and inhomogeneous were offered. When each sample is considered individually, there were only two attributes, thick/creamy and glossy, which were significantly different compared to other attributes for all samples (more than 50% responses). Differences between samples for given attributes are only discussed for groups of samples of the same placebo, i.e., P1 and samples with lipid extracts; P3 and samples with ethanol extracts. For attribute thin/milky, a significant difference was found only between the SEE and P3 samples, P3 was described as significantly thinner than SEE which is consistent with the results of the rheological measurements. Significant differences were found in the color of the samples, showing that the addition of extract significantly affects the color of the product.

**Figure 7:**
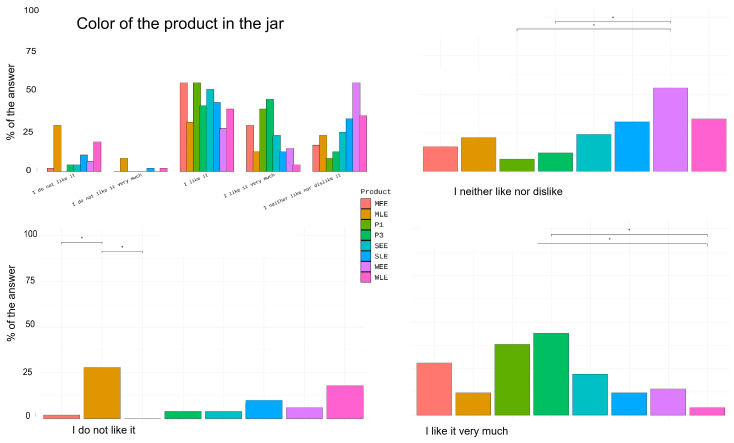
CATA results of ‘Color of the product in the jar’ for attributes ‘I like it very much’, ‘I like it’, ‘I neither like nor dislike it’, ‘I do not like it’, and ‘I do not like it very much’ presented in percentage of answers; with attributes for which statistically significant differences between the samples were obtained; * statistically significant differences, *p* < 0.05.

One of the domains investigated was whether users liked the color of the product (Figure 7). There are a number of significant differences between the answers provided for each sample. For example, there was a significant difference for P1 sample between ‘I like it very much’ vs. ‘I neither like nor dislike it’, or for WLE between ‘I like it very much’ vs. ‘I like it’, etc. The results show that some users may like or dislike the color of the same product more or less and therefore we did not consider these results to be significant. More important is the comparison between the samples. Users liked P1 very much and significantly more than WLE, while they disliked the MLE sample significantly more than P1. The MLE sample was with the most ‘I do not like it’ and ‘I do not like it very much’ responses. On the other hand, the results show that there are also users who liked its color very much. In samples with ethanol extracts only, for ‘I neither like it nor dislike it’, there was a significant difference between P3 and WEE. These results could indicate that the maize lipid extract gives the product a color that could be a limiting factor for its commercialization in topical products.

Regarding the intensity of product smell, the majority of panelists rated the products with extracts as products whose smell they perceived as ‘slightly felt’ and ‘felt’. Only the MLE sample was significantly marked as a sample that is ‘strongly felt’, the significance was reached when compared to P1, WLE, and SLE (Figure S3). Similar to the color of the product, the perception of the smell (‘feeling about product smell’) was also rated differently between ‘pleasant’ and ‘very unpleasant’ (Figure S4). There were statistically significant differences only among the attributes for each sample but not between samples. All samples were rated significantly more often as ‘pleasant’ compared to ‘unpleasant’ and ‘very unpleasant’, with the exception of the MLE sample.

In Figure S5, results obtained for the skin feel during product application and 3 min after are presented. All samples significantly spread easily/glide on the skin, moisturize the skin, make the skin smooth, leave a light feeling on the skin, are easy to rub in, and are quickly absorbed by the skin. In general, all samples showed a similar skin feel during the application, which was to be expected based on conducted instrumental measurements. For the after-feel phase, the skin was described mainly as smooth, soft, and shiny after each product application.

There were no significant differences in the answers to the question about the purchase of products, either within the individual samples or between all samples (Figure S6). The highest percentage of answers ‘for sure’ was given to the MEE sample, ‘probably’ to P1 and MEE samples, and ‘I probably wouldn’t’ and ‘I certainly wouldn’t’ to MLE. It can be observed that the sample with the maize lipid extract has the lowest probability of purchase. The analysis did not confirm the existence of a significant correlation between the color or smell of the product and the eventual purchase decision.

The sensory evaluation showed that the extracts induced significant changes in the color and smell of the placebo formulations, while the skin feel and sensation after application remained similar. The results of the sensory analysis for the attributes perceived by sight agree well with the instrumental measurements. This is interesting because it shows that the sense of sight can also distinguish the features that we perceive with the sense of touch or with an instrument. Regarding color and smell, the results show that the same color can be very liked by one person and not by another, and that the smell can be pleasant for someone and unpleasant for another. Of the extracts studied, only the maize lipid extract gave the product a color and smell that was perceived as unpleasant by a significant number of panelists. This could be a limiting factor for its commercialization in topical products. However, a certain number of panelists liked the MLE color very much and found the smell pleasant. This emphasizes the importance of product claims when it comes to natural ingredients in topical products [58]. It is very important to inform consumers about what they can expect when they buy such products. The smell of the product is the most important feature that determines consumer purchasing behavior [74]. Since the results obtained show that the same smell can be liked a lot by one person and not by another, it is important to create good profiles of the target groups so that the smell of the product is not a limiting factor. The statistical analysis showed no significant correlation between the color or smell of the product and the eventual purchase decision, but the sensory evaluation showed that the sample with the maize lipid extract, which had the strongest influence on the smell and color of the placebo cream, had the lowest probability of purchase.

**Figure 8:**
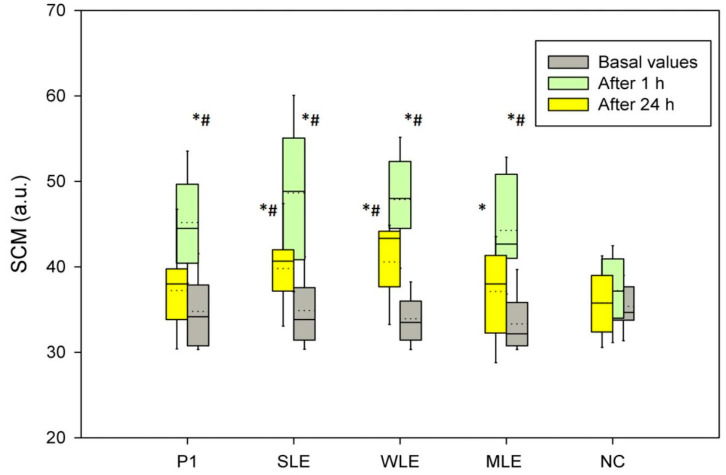
Stratum corneum moisturization (SCM) initially (basal values), after 1 h, and after 24 h; * statistically significant changes compared to basal values, # statistically significant changes compared to non-treated control (*p* < 0.05).

To investigate the possible contribution of extracts to the short-term efficacy of products on the skin, formulations with lipid extracts were selected. Stratum corneum moisturization (SCM) and transepidermal water loss (TEWL) were measured. TEWL was initially measured before and 24 h after application of the sample. There were no differences in TEWL values 24 h after any sample application compared to basal values or non-treated control. SCM values were measured at baseline, 1 h, and 24 h after product application (Figure 8). SCM was significantly increased 1 h after the application of all samples compared to basal values and non-treated control (NC). After 24 h, SCM was still significantly increased after application of SLE, MLE, and WLE compared to basal values, but only for SLE and WLE compared to non-treated control. These results are consistent with the in vitro skin occlusivity results.

Since the TEWL, which is a sensitive indicator of skin irritation, did not change in the in vivo study, it can be said that topical products with lipid extracts do not lead to a disruption of the skin barrier after a single application [75]. Likewise, their addition to the cream increases the moisturizing effect of the cream. These results are significant from the safety aspect of the ingredients and, together with the cytotoxicity test, indicate their good safety profile. The fact that they contribute to the moisturizing effect with the results of the skin occlusivity test confirms their skin conditioning—emollient effect. Due to the composition of the fatty acids of the lipid extracts, a more significant contribution of creams with lipid extracts to the integrity of the skin barrier can be assumed with long-term use.

## 4. Conclusions

Through systematic investigations and experiments, this research aims to demonstrate the potential of maize, wheat, and sunflower waste extracts as versatile raw materials for the development of topical products. Although their addition influences the physical and chemical properties of the placebo cream, the basic structure is not affected. The extracts tested have a satisfactory safety profile and even have a positive effect on the viability of human keratinocytes, but attention must be paid to their concentrations and the overall safety of the final product. Lipid extracts have the potential to be natural structural components of the lipid phase and good co-stabilizers for modern emulsifiers. All products derived from crop residues can contribute to the microbiological quality of products and have the potential to be used as multifunctional ingredients in self-preserving formulations. The investigated extracts contribute to the efficacy of topical products, primarily to the emollient effect, which consequently leads to an increase in skin moisture. However, these natural ingredients have a significant impact on important sensory properties such as smell and color, which can have both positive and negative effects on consumer acceptance and must be taken into account in the formulation.

The use of maize, wheat, and sunflower waste as raw materials for topical products represents a valorization of waste and is in line with broader sustainability goals by addressing key challenges such as waste reduction, resource conservation, and carbon footprint reduction. By bridging the gap between agricultural waste valorization and product development, this study aims to contribute to the realization of a more sustainable and resilient future for the global skincare and healthcare sector.

## Figures and Tables

**Table 1:** Composition of ethanol extracts.

	Ethanol Maize Extract	Ethanol Wheat Extract	Ethanol Sunflower Extract
Total phenolic content (mg of gallic acid equivalents (GAE) per g)	20.44 mg GAE/g	23.19 mg GAE/g	15.83 mg GAE/g
Total flavonoid content (mg of quercetin equivalents (QE) per g)	9.37 mg QE/g	15.22 mg QE/g	8.98 mg QE/g
Isolated compounds	*p*-Coumaric acid = 1.64 mg/g Ferulic acid = 1.21 mg/g Tricin = 1.99 mg/g	*p*-Coumaric acid = 2.14 mg/g Tricin = 3.92 mg/g	Chlorogenic acid = 2.40 mg/g

**Table 2:** Composition of lipid extracts.

Fatty Acids	Lipid Wheat Extract (%)	Lipid Maize Extract (%)	Lipid Sunflower Extract (%)
C14:0	1.44 ± 0.31	0.49 ± 0.02	nd
C16:0	23.74 ± 8.23	11.84 ± 0.46	6.13 ± 0.28
C16:1	nd	4.61 ± 0.10	nd
C18:0	3.10 ± 0.79	2.58 ± 0.08	2.56 ± 0.08
C18:1	12.93 ± 3.99	29.32 ± 0.30	26.54 ± 1.02
C18:2	26.76 ± 7.61	24.01 ± 0.21	34.34 ± 0.97
C18:3n3	1.59 ± 0.48	2.93 ± 0.02	0.88 ± 0.12
C20:0	6.97 ± 2.94	4.37 ± 0.08	5.53 ± 0.53
C22:0	6.92 ± 3.30	4.74 ± 0.36	4.84 ± 0.20
C24:0	3.16 ± 0.15	2.10 ± 0.25	2.33 ± 0.18
Other components *	21.68 ± 6.96	13.02 ± 0.10	16.85 ± 1.97

nd—not detected. * 21.68%, 13.02%, and 16.85% of lipid extracts of wheat, maize, and sunflower, respectively, where other components are different chemical compounds (fatty acids, fatty alcohols, aldehydes, ketones, sterols, and waxes).

**Table 3:** Composition of final/investigated formulations.

Ingredients (INCI) [% wt]	P1	P3	WLE	MLE	SLE	WEE	MEE	SEE
Polyglyceryl-6 distearate, jojoba esters, polyglyceryl-3 beeswax, and cetyl alcohol	4.0	4.0	4.0	4.0	4.0	4.0	4.0	4.0
Brassica glycerides	3.0	1.0	2.0	2.0	2.0	1.0	1.0	1.0
Caprylic/capric triglyceride	20.0	20.0	20.0	20.0	20.0	20.0	20.0	20.0
Triticum aestivum lipid extract (wheat lipid extract—LE-W)	/	/	2.0	/	/	/	/	/
Triticum aestivum ethanol extract (wheat ethanol extract—EE-W)	/	/	/	/	/	0.1	/	/
Zea mays lipid extract (maize lipid extract—LE-M)	/	/	/	2.0	/	/	/	/
Zea mays ethanol extract (maize ethanol extract—EE-M	/	/	/	/	/	/	0.1	/
Helianthus annuus lipid extract (sunflower lipid extract—LE-S)	/	/	/	/	2.0	/	/	/
Helianthus annuus ethanol extract (sunflower ethanol extract—EE-S	/	/	/	/	/	/	/	0.1
Phenoxyethanol and ethylhexylglycerol	0.8	0.8	0.8	0.8	0.8	0.8	0.8	0.8
Aqua/Water: up to	100.0	100.0	100.0	100.0	100.0	100.0	100.0	100.0

**Table 4:** pH and conductivity measurements of creams, initially (after 7 days) and after 6 months of storage at room temperature with 90% confidence interval for pH values.

Sample	pH ± SD		90% Confidence Interval ^#^	Conductivity ± SD (μS/cm)	
	After 7 Days	After 6 Months		After 7 Days	After 6 Months
P1	7.35 ± 0.05	7.09 ± 0.03 *	8.09–6.62	13.87 ± 0.28	14.63 ± 0.42
WLE	7.26 ± 0.06	6.65 ± 0.05	7.98–6.53	46.00 ± 0.88	20.09 ± 3.40 *
MLE	7.06 ± 0.07	6.72 ± 0.06 *	7.77–6.35	56.00 ± 1.22	45.63 ± 0.45 *
SLE	6.74 ± 0.05	6.67 ± 0.04	7.42–6.07	64.00 ± 1.57	56.03 ± 0.55
P3	7.06 ± 0.01	7.35 ± 0.03 *	7.77–6.35	31.01 ± 1.94	23.87 ± 0.12
WEE	6.76 ± 0.02	7.26 ± 0.02 *	7.44–6.11	83.30 ± 1.73	76.11 ± 0.87 *
MEE	6.79 ± 0.01	7.06 ± 0.03 *	7.47–6.11	135.68 ± 4.81	126.24 ± 2.32 *
SEE	6.49 ± 0.02	6.74 ± 0.01 *	7.14–5.84	115.10 ± 5.45	104.15 ± 1.32 *

* Statistically significant changes compared to initial values (after 7 days). ^#^ The interval was recalculated in relation to the pH values after 7 days.

**Table 5:** Diameter of emulsion droplets of investigated creams.

Sample	Dimensions (μm)		
	Mean ± SD	Minimum	Maximum
P1	39.31 ± 27.76	9.49	99.77
WLE	12.90 ± 2.88	3.07	26.84
MLE	11.94 ± 8.37	3.43	40.38
SLE	11.11 ± 8.14	2.75	42.86
P3	12.71 ± 10.44	1.44	78.74
WEE	9.68 ± 8.17	1.14	56.24
MEE	7.38 ± 4.07	0.51	33.62
SEE	10.96 ± 9.22	0.72	110.14

**Table 6:** Apparent maximum (η_max_) and minimum (η_min_) viscosities of investigated creams.

Sample	η_max_ Initially (Pa·s)	η_max_ after 6 Months (Pa·s)	η_min_ Initially (Pa·s)	η_min_ after 6 Months (Pa·s)
P1	3.84	6.24	0.395	0.657
WLE	2.28	3.78	0.150	0.285
MLE	4.52	3.79	0.242	0.275
SLE	3.74	3.96	0.289	0.319
P3	2.19	2.71	0.191	0.179
WEE	3.09	5.50	0.216	0.275
MEE	5.07	6.98	0.267	0.303
SEE	5.92	8.4	0.287	0.306

## Data Availability

The original contributions presented in the study are included in the article/Supplementary Material, further inquiries can be directed to the corresponding author.

## Supplementary Materials

The following supporting information can be downloaded at https://www.mdpi.com/article/10.3390/pharmaceutics16091182/s1: Figure S1: Stickiness and water washability of investigated creams presented as amount of the sample left on cotton wool (%) for stickiness and washed amount of sample (%) for washability; Figure S2: Overall CATA results of ’Look of the product in jar’ for attributes: thick/creamy, colored/not white, glossy, matt/not glossy, thin/milky grainy, inhomogeneous with attributes for which statistically significant differences between the samples were obtained; Figure S3: CATA results of ‘Intensity of the products smell’ (How do you rate the smell of the product) for attributes: odorless, slightly felt, felt, strongly felt, intense smell presented in percentage of answers; with attributes for which statistically significant differences between the samples were obtained; Figure S4: Overall CATA results of ’Feeling about product smell’ for attributes: very unpleasant, unpleasant, neither pleasant nor unpleasant and pleasant; Figure S5: Overall CATA results of Skin feel during product application for attributes: spreads easily/glides on the skin, moisturizes the skin, oils the skin, makes the skin smooth, makes the skin sticky, leaves a light feeling on the skin, leaves a heavy feeling on the skin, hard to spread; thick/creamy, thin, hard to rub in, easy to rub in, it is quickly absorbed by the skin, it is slowly absorbed by the skin and for ’Skin feel after 3 minutes’ for attributes: smooth, soft, tender, sticky, shiny, dry, tight, oily with attributes for which statistically significant differences between the samples were obtained; Figure S6: CATA results of ‘Intention to buy the product if it was on the market minutes’ for attributes: for sure; probably; maybe I would, maybe I wouldn’t; I probably wouldn’t; I certainly wouldn’t, presented in percentage of answers; Table S1: Texture parameters of investigated creams.
