# Supplementary material for: Extracts from Wheat, Maize, and Sunflower Waste as Natural Raw Materials for Cosmetics: Value-Added Products Reaching Sustainability Goals
Source: Pharmaceutics. 2024 Sep 7;16(9):1182. doi: 10.3390/pharmaceutics16091182 (PMC11435005; doi:10.3390/pharmaceutics16091182)

Table S1. Texture parameters of investigated creams.

Sample	Firmness [gf]	Cohesiveness [gf]	Consistency [gfs]	Index of viscosity [gfs]
**P1**	18.59 ± 0.86	3.63 ± 0.14	34.47 ± 1.32	15.47 ± 0.98
**WLE**	6.15 ± 0.10	1.50 ± 0.07	15.46 ± 0.91	6.80 ± 0.65
**MLE**	9.13 ± 0.09	2.11 ± 0.19	18.46 ± 1.14	9.34 ± 1.14
**SLE**	10.74 ± 0.33	2.48 ± 0.08	29.47 ± 2.06	10.03 ± 1.31
**P3**	6.54 ± 0.33	1.51 ± 0.18	17.65 ± 0.87	6.72 ± 0.66
**WEE**	6.71 ± 0.39	1.96 ± 0.18	15.55 ± 1.11	7.28 ± 0.81
**MEE**	7.83 ± 0.30	1.80 ± 0.17	21.47 ± 1.33	7.47 ± 1.06
**SEE**	8.41 ± 0.26	1.33 ± 0.06	19.32 ± 0.74	5.98 ± 0.43

Figure S1. Stickiness and water washability of investigated creams presented as amount of the sample left on cotton wool (%) for stickiness and washed amount of sample (%) for washability.

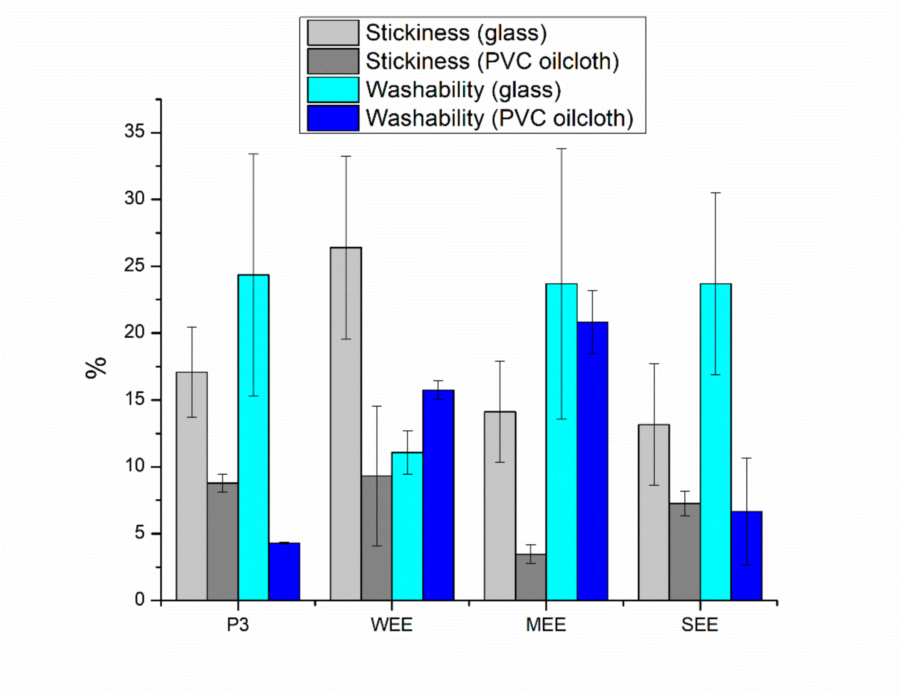

Figure S2: Overall CATA results of 'Look of the product in jar' for attributes: thick/creamy, colored/not white, glossy, matt/not glossy, thin/milky grainy, inhomogeneous with attributes for which statistically significant differences between the samples were obtained; * statistically significant differences, $p < 0.05$.

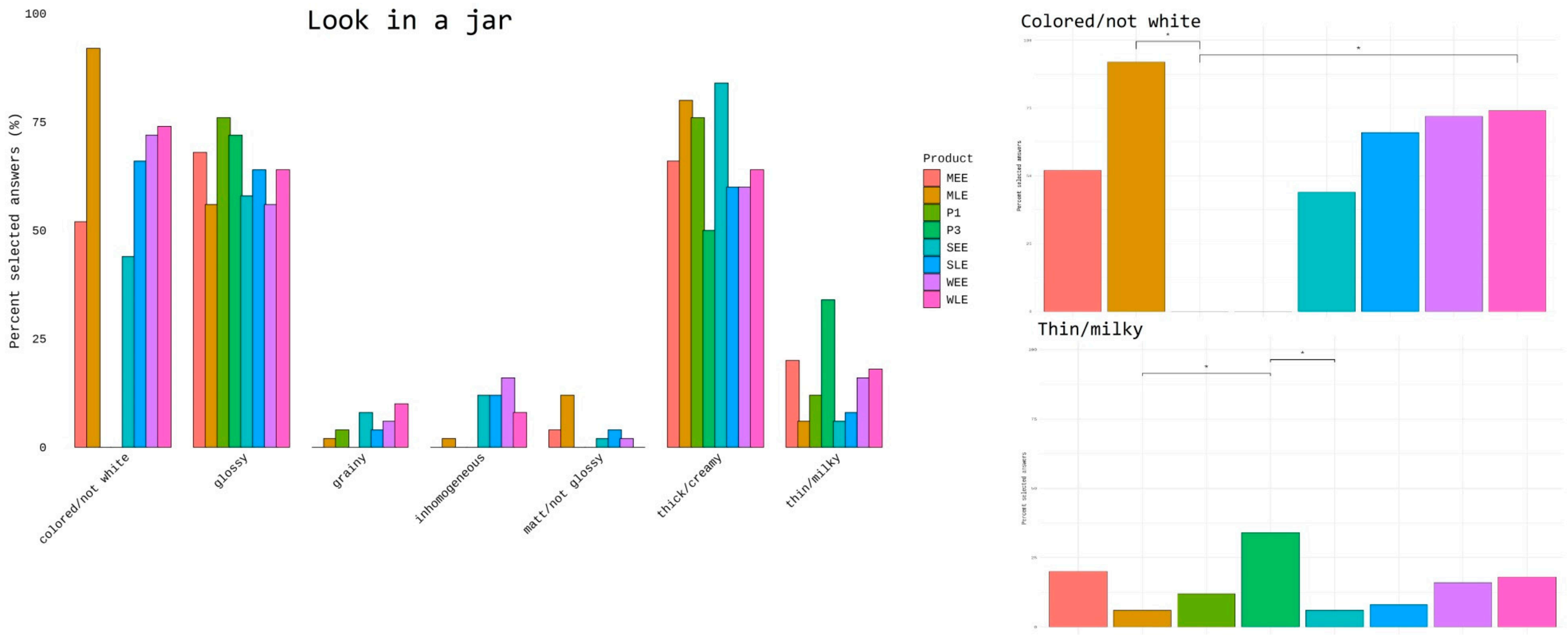

Figure S3. CATA results of 'Intensity of the products smell' (How do you rate the smell of the product) for attributes: odorless, slightly felt, felt, strongly felt, intense smell presented in percentage of answers; with attributes for which statistically significant differences between the samples were obtained; * statistically significant differences, $p < 0.05$.

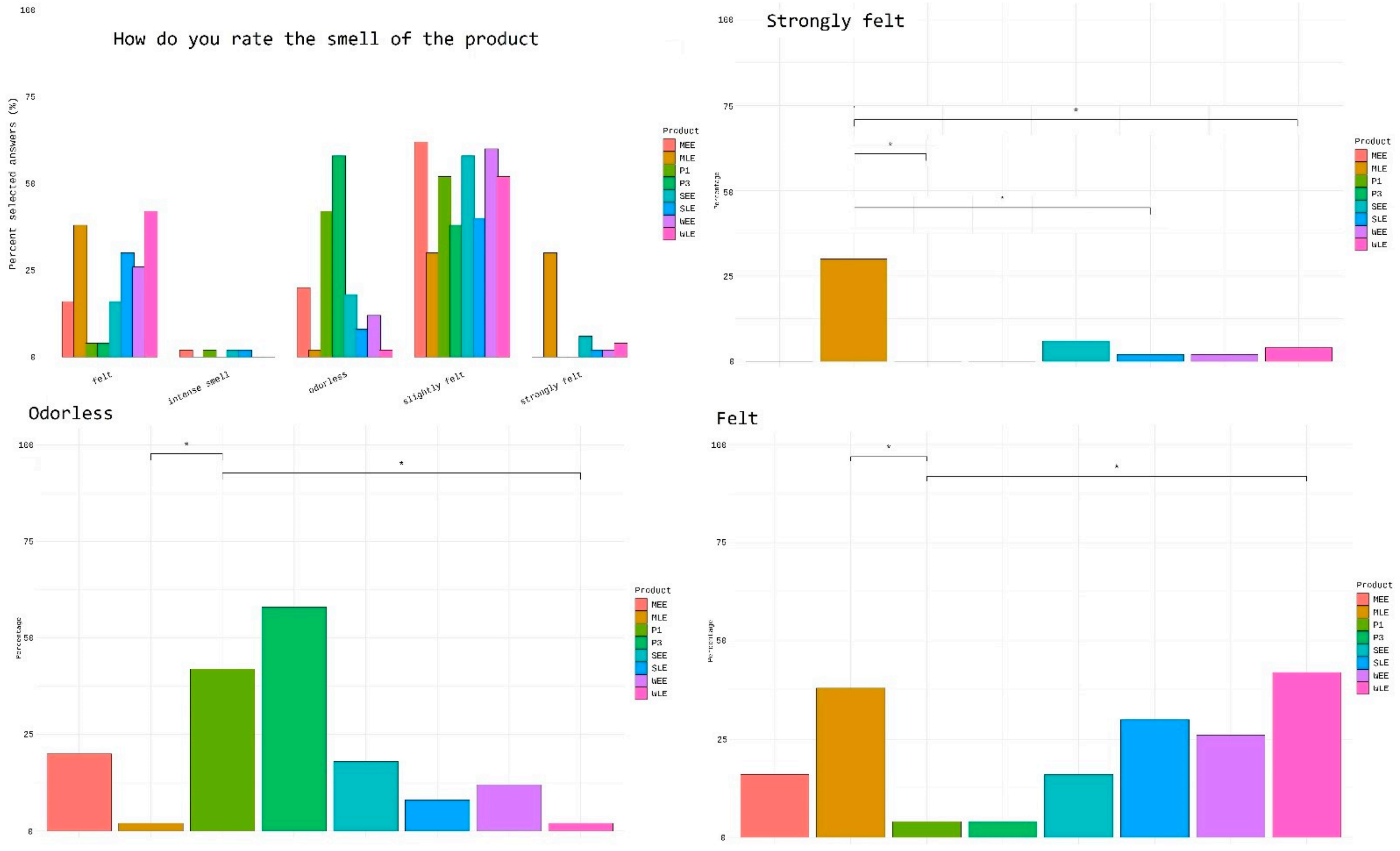

Figure S4: Overall CATA results of ’Feeling about product smell’ for attributes: very unpleasant, unpleasant, neither pleasant nor unpleasant and pleasant.

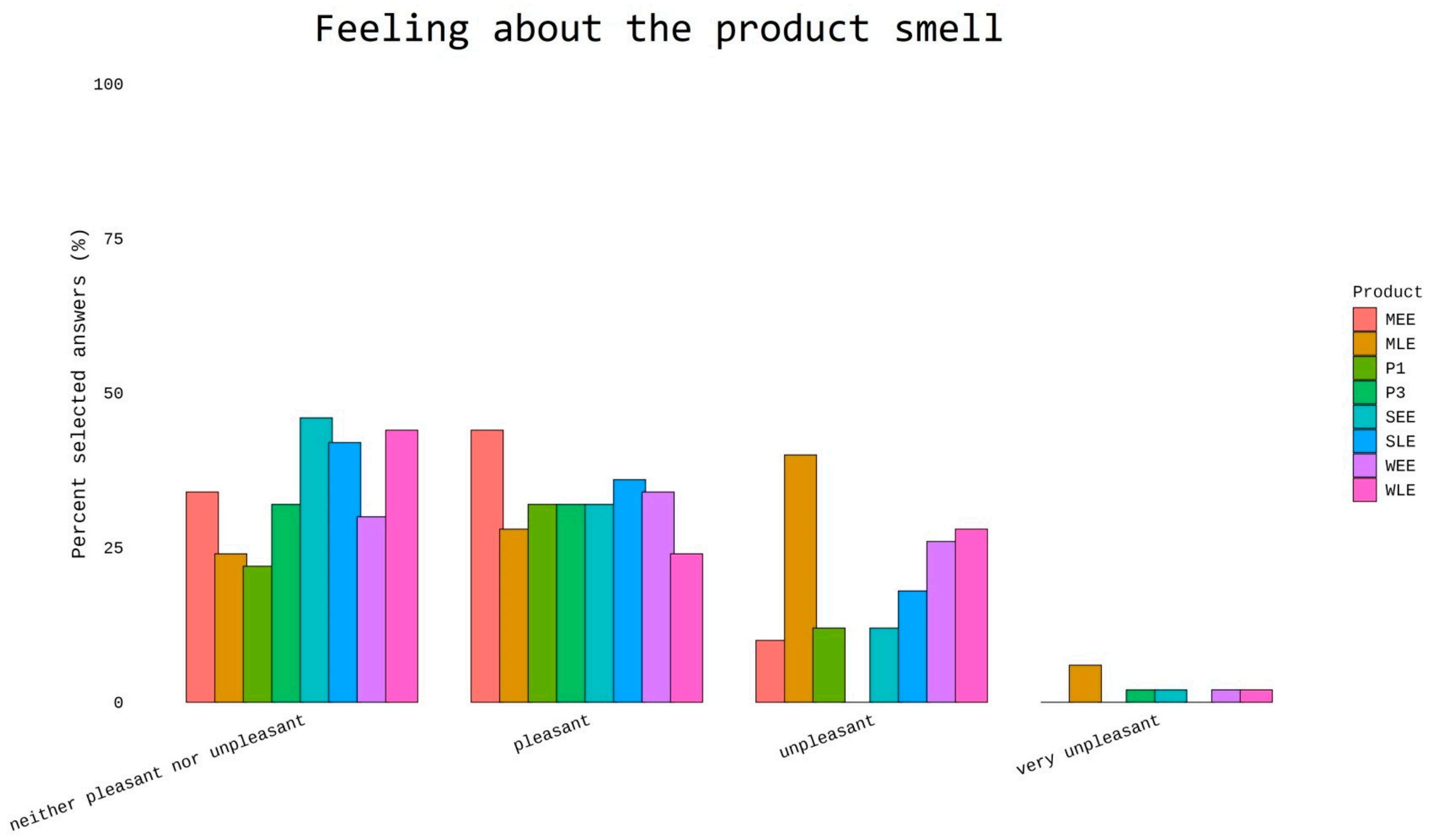

Figure S5: Overall CATA results of Skin feel during product application for attributes: spreads easily/glides on the skin, moisturizes the skin, oils the skin, makes the skin smooth, makes the skin sticky, leaves a light feeling on the skin, leaves a heavy feeling on the skin, hard to spread; thick/creamy, thin, hard to rub in, easy to rub in, it is quickly absorbed by the skin, it is slowly absorbed by the skin and for 'Skin feel after 3 minutes' for attributes: smooth, soft, tender, sticky, shiny, dry, tight, oily with attributes for which statistically significant differences between the samples were obtained; * statistically significant differences, $p < 0.05$.

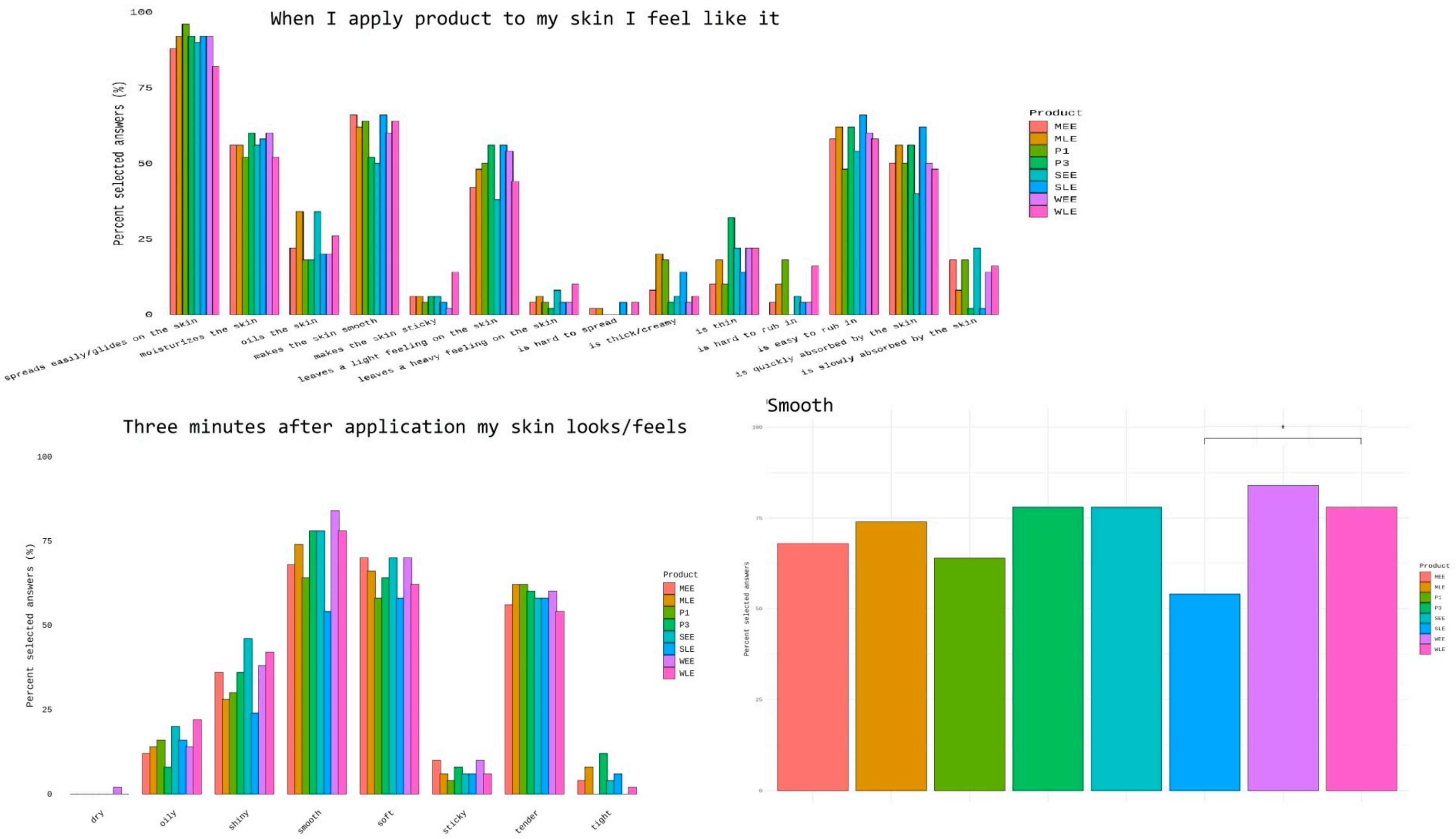

Figure S6. CATA results of 'Intention to buy the product if it was on the market minutes' for attributes: for sure; probably; maybe I would, maybe I wouldn't; I probably wouldn't; I certainly wouldn't, presented in percentage of answers.

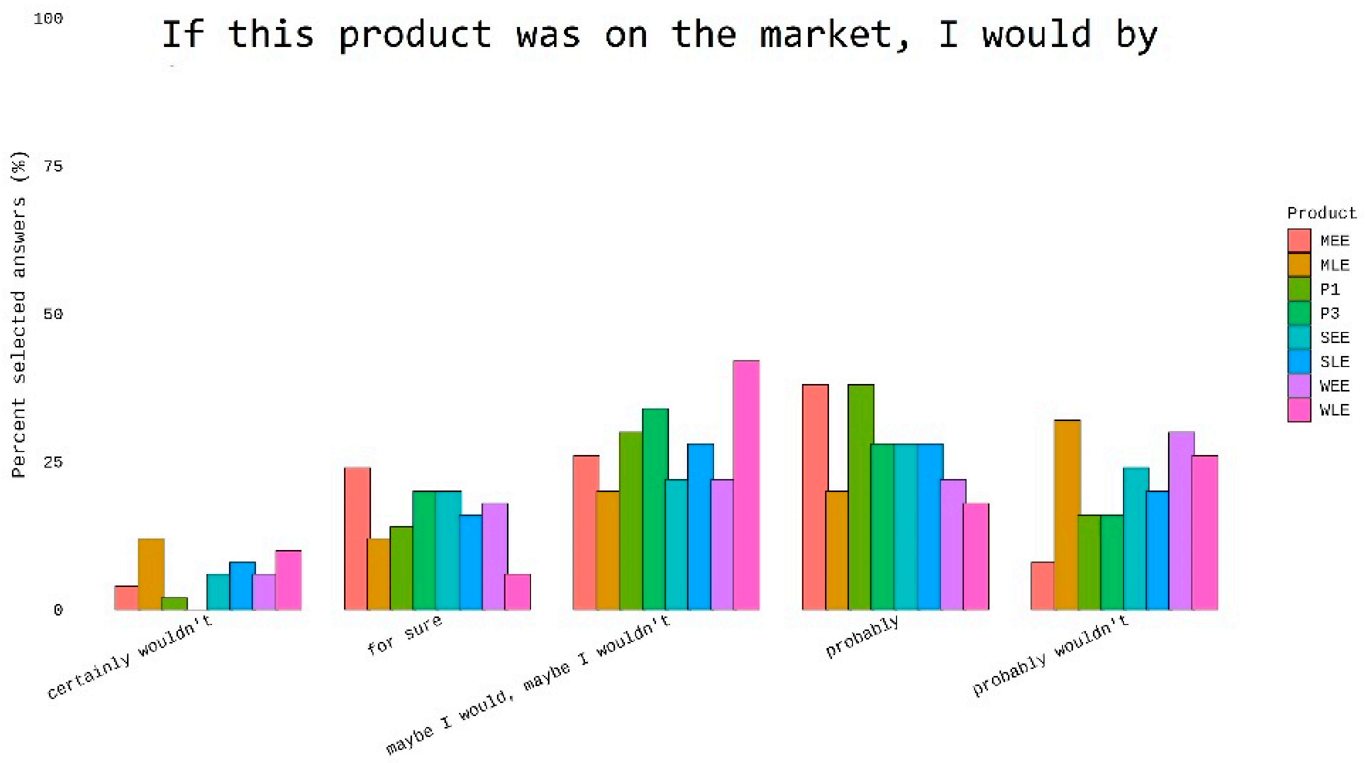

Supplement: Supplementary file 1 [file pharmaceutics-16-01182-s001.zip › pharmaceutics-3122211-supplementary.pdf]
